# Physician and Biomedical Scientist Harassment on Social Media During the COVID-19 Pandemic

**DOI:** 10.1001/jamanetworkopen.2023.18315

**Published:** 2023-06-14

**Authors:** Regina Royan, Tricia Rae Pendergrast, Nicole C. Woitowich, N. Seth Trueger, Lawren Wooten, Shikha Jain, Vineet M. Arora

**Affiliations:** 1 Department of Emergency Medicine, Northwestern University Feinberg School of Medicine, Chicago, Illinois; 2 Assistant Editor, *JAMA Network Open*, Chicago, Illinois; 3 Northwestern University Feinberg School of Medicine, Chicago, Illinois; 4 Department of Medical Social Sciences, Northwestern University Feinberg School of Medicine, Chicago, Illinois; 5 Department of Pediatrics, University of California, San Francisco, San Francisco; 6 Division of Hematology and Oncology, Department of Medicine, University of Illinois, Chicago; 7 Department of Medicine, University of Chicago Pritzker School of Medicine, Chicago; 8 Digital Media Editor, *JAMA Network Open*, Chicago, Illinois

## Abstract

This survey study assesses the frequency and nature of harassment on social media experienced by physicians, biomedical scientists, and trainees during the COVID-19 pandemic.

## Introduction

Advocacy on social media is not without risks. In a survey conducted before the COVID-19 pandemic, 23.3% of physicians reported personal attacks on social media, primarily for public health advocacy on topics including firearms, vaccinations, and abortion access.^1^ While the Surgeon General^2^ encourages physicians and scientists to use social media to address misinformation,^3^ concerns for harassment remain.^4,5,6^

To our knowledge, no study has examined online harassment of physicians and scientists during the pandemic. We surveyed physicians, biomedical scientists, and trainees who experienced online harassment during the pandemic, particularly relating to dissemination of COVID-19 public health information.

## Methods

Northwestern University Institutional Review Board deemed this survey study exempt from review and informed consent because all responses were anonymous. We followed AAPOR reporting guidelines.

Survey design mirrored a prior study^1^ and used a collaborative consensus process to develop questions regarding online harassment. Participants were recruited through Twitter using a standardized message (eFigure in Supplement 1). Inclusion criteria included US residence and self-reported profession as a physician, biomedical scientist, or trainee. Participants self-reported demographic information on age, gender, and race and ethnicity. Responses were collected from July 18 to August 21, 2022 (eMethods in Supplement 1). Comparative statistics were calculated with 2-tailed χ^2^ analyses using Stata, version 17.0 (StataCorp LLC), with *P* < .05 considered significant.

## Results

Of 1028 survey views, 359 respondents met the inclusion criteria. Most respondents (120 [33%]) were aged 35 to 44 years (203 females [57%], 140 males [39%], and 16 [4%] identified as transgender male or man, transgender female or woman, gender nonbinary, or self-described gender). In all, 238 respondents (66%) reported harassment on social media (Table 1). Of these individuals, 210 (88%) reported harassment due to advocacy, 107 (45%) reported harassment on the basis of gender, 65 (27%) race or ethnicity, 31 (13%) sexual orientation, 15 (6%) due to disability, and 74 (31%) due to other self-described reasons. Women and other genders were more likely than men to report harassment based on gender (88 [67%] and 7 [58%] vs 12 [13%], respectively; *P* < .001). Additionally, 9 of 11 Black respondents (82%) reported harassment based on race or ethnicity vs 14 of 27 Asian respondents (52%) and 26 of 174 White respondents (15%) (*P* < .001). Harassment based on race or ethnicity was reported by 9 of 13 (69%) Hispanic respondents vs 56 of 225 (25%) non-Hispanic respondents (*P* < .001).

**Table 1.** Respondents Reporting Harassment on Social Media

Variable	Any online harassment		Harassment regarding gender		Harassment regarding race or ethnicity		Harassment regarding advocacy		Sexual harassment		Harassment regarding comments on COVID-19	
	Respondents, No./total No. (%)	*P* value	Respondents, No./total No. (%)	*P* value	Respondents, No./total No. (%)	*P* value	Respondents, No./total No. (%)	*P* value	Respondents, No./total No. (%)	*P* value	Respondents, No./total No. (%)	*P* value
No. reporting harassment/total No. eligible for participation	238/359 (66)	NA	107/238 (45)	NA	65/238 (27)	NA	210/238 (88)	NA	111/359 (31)	NA	228/359 (64)	NA
Age, y												
18-24	1/2 (50)	.45	1/1 (100)	.009	0/1	.24	1/1 (100)	.14	1/2 (50)	.13	2/2 (100)^a^	.26
25-34	49/80 (61)		32/49 (65)		18/49 (37)		40/49 (82)		34/80 (43)		46/80 (58)	
35-44	79/120 (66)		32/79 (41)		25/79 (32)		76/79 (96)		36/120 (30)		74/120 (62)	
45-54	64/92 (70)		23/64 (36)		11/64 (17)		55/64 (86)		25/92 (27)		61/92 (66)	
55-64	41/56 (73)		19/41 (46)		10/41 (24)		35/41 (85)		14/56 (25)		41/56 (73)	
>65	4/9 (44)		0/4		1/4 (25)		3/4 (75)		1/9 (11)		4/9 (44)	
Gender												
Female	132/203 (65)	.69	88/132 (67)	<.001	37/132 (28)	.82	113/132 (86)	.37	84/203 (41)	<.001	122/203 (60)	.31
Male	94/140 (67)		12/94 (13)		24/94 (26)		86/94 (91)		19/140 (14)		95/140 (68)	
Other^b^	12/16 (75)		7/12 (58)		4/12 (33)		11/12 (92)		8/16 (50)		11/16 (69)	
Race												
Asian	27/41 (66)	.63	13/27 (48)	.60	14/27 (52)	<.001	26/27 (96)	.24	15/41 (37)	.70	25/41 (61)	.57
Black	11/16 (69)		7/11 (64)		9/11 (82)		8/11 (73)		6/16 (38)		8/16 (50)	
White	174/257 (68)		76/174 (44)		26/174 (15)		153/174 (88)		75/257 (29)		168/257 (65)	
Other^c^	26/45 (58)		11/26 (42)		16/26 (62)		23/26 (88)		15/45 (33)		27/45 (60)	
Ethnicity^d^												
Hispanic	13/21 (62)	.66	5/13 (38)	.63	9/13 (69)	<.001	11/13 (85)	.68	8/21 (38)	.46	10/21 (48)	.12
Non-Hispanic	225/338 (67)		102/225 (45)		56/225 (25)		199/225 (88)		103/338 (30)		218/338 (65)	
Identify as having a disability												
No	221/335 (66)	.63	94/221 (43)	.007	60/221 (27)	.84	195/221 (88)	.99	98/335 (29)	.01	209/335 (62)	.09
Yes	17/24 (71)		13/17 (76)		5/17 (29)		15/17 (88)		13/24 (54)		19/24 (79)	
Identify as minority on basis of gender or sexuality												
No	185/282 (66)	.60	74/185 (40)	.004	42/185 (23)	.003	158/185 (85)	.01	74/282 (26)	<.001	178/282 (63)	.77
Yes	53/77 (69)		33/53 (62)		23/53 (43)		52/53 (98)		37/77 (48)		50/77 (65)	
Trainee												
No	201/290 (69)	.01	81/201 (40)	.001	50/201 (25)	.05	178/201 (89)	.72	84/290 (29)	.10	195/290 (67)	.003
Yes	37/69 (54)		26/37 (70)		15/37 (41)		32/37 (86)		27/69 (39)		33/69 (48)	
Use social media to post public health messages												
No	18/46 (39)	<.001	6/18 (33)	.30	6/18 (33)	.55	12/18 (67)	.003	4/46 (9)	<.001	13/46 (28)	<.001
Yes	220/313 (70)		101/220 (46)		59/220 (27)		198/220 (90)		107/314 (34)		215/313 (69)	
Verified profile on social media^e^												
No	199/314 (63)	.002	89/199 (45)	.87	51/199 (26)	.19	173/199 (87)	.16	93/313 (30)	.16	189/314 (60)	.001
Yes	39/45 (87)		18/39 (46)		14/39 (36)		37/39 (95)		18/45 (40)		39/45 (87)	

Abbreviation: NA, not applicable.

^a^ One respondent selected *no* when asked about online harassment, but later stated they were harassed with respect to their comments on COVID-19.

^b^ Other gender includes transgender male or man, transgender female or woman, gender nonbinary, or self-described gender. Nationally, practicing physicians are 35.9% female and 64.1% male (American Association of Medical Colleges. Diversity in Medicine Facts and Figures 2019).

^c^ Other race includes multiracial, Native Hawaiian or Pacific Islander, or self-described. In the US, 0.1% practicing physicians are American Indian or Alaska Native, 63.9% are White, 19.2% are Asian, 3.6% are Black or African American, 5.5% are Hispanic, 2.0% are multiracial, and 5.6% other race and ethnicity (American Association of Medical Colleges. Diversity in Medicine Facts and Figures 2019).

^d^ Respondents selected from 2 choices for ethnicity: Hispanic or non-Hispanic.

^e^ Survey completed prior to verification policy changes at on the social media platform.

Of 359 respondents, 228 (64%) reported harassment related to comments made about the COVID-19 pandemic, 111 (31%) reported being sexually harassed, and 66 (18%) reported their private information had been shared (ie, doxxing). One hundred forty-four respondents provided open-ended responses. Representative themes of harassment are shown in Table 2, which highlights extreme threats and impacts on mental health. A total of 228 of 359 participants (64%) reporting any online harassment reported the pandemic changed the way that they use social media. Those using social media to post public health messages were more likely than those who did not to report online harassment (220 of 313 [70%] vs 18 of 46 [39%]; *P* < .001).

**Table 2.** Themes Identified From Respondents’ Comments and Advocacy Topics That Resulted In Harassment

Themes	No. of responses	Representative example
Forms of harassment^a^		
Doxxing^b^	28	“I posted on LinkedIn about the safety of the COVID vaccine and masking and refuted others who posted misinformation, and I was reported to my company[’s] [human resources] department.”
		“I have had my office address posted, have had people leave false reviews, have received death threats, have received harassment related to social media at my home address, and have had people repeatedly try to have my job terminated or cause harm to my career.”
		“My Healthgrades [profile is] full of bogus negative reviews from antivaxxers.”
		“I’ve been doxxed several times, including fake social media accounts using my unique name - on multiple accounts on social media (and even Pornhub with my photos mixed in w[ith] a nude model that has an uncanny resemblance to me); fake reports to medical board; fake reviews on physician rating sites claiming I’m racist against white people.”
Impacts to mental health	11	“I use social media less. I found it was too draining for me and my mental health was suffering.”
		“I’ve had to take a step back from a lot of health discourse. This constant battle is taking a lot out of me. Unfortunately, I tend to now stay out of it.”
Fear or threats of violence	13	“Would be very reluctant using public account to take controversial positions as I don’t want work censure or to be harassed at home or in my private life. I have small kids at home to protect.”
		“I have over 30 threats to rape, kill, or assault me posted to Twitter, Reddit, and private websites. I have lost count of the number of law enforcement reports I have made, but the accounts that make the most direct threats are always anonymous.”
		“I have semi-routinely been harass[ed] with violent threats (including menacing messages (“we’ll see you soon,” “your time will come”), and pictures (such as images of individuals being hung) as well as sexual harassment (lewd images, advances, and threats). We have involved the police in some situations. What is most disturbing to me, is that many of these individuals are local and regularly show up to protest at my workplace…my hospital has allowed them to be on hospital property to protest and offered very minimal protection for me.”
		“Repeated death threats to myself and coworkers for years posted online and sent in direct messages. Family including children threatened, leading to law enforcement intervention and limitation of activities at school for safety. Harassment at home and work, stalked at lectures across the country, and physically assaulted twice.”
Disappointment in response	13	“Being harassed on social media was one of the worst—if not the worst—experience(s) of my career and of my life. I have questioned myself and my work. I discovered that when I needed support—no one was there to stand up for me—no one. I am grieving for what I have lost—my reputation, my friends, my colleagues.”
		“The harassment is out of control and social media platforms don’t seem to care, and don’t... protect us in any way.”
Sexual harassment	11	“I’ve been doxxed several times including fake social media accounts... [on] Pornhub with my photos mixed in w[ith] a nude model.”
		“There is a constant stream of sexual messages. Most of the[m] I can screen out, but I get unwanted messages at least once a week. I don’t engage, which usually works, but I don’t check my DMs now because of this.”
Gender	5	“Someone posted my image on Twitter/Facebook without my consent and discussed my gender presentation.”
		“There were... messages that described in detail what the people... wanted to do to me and other trans people. Many of which were sexual or violent in nature, or both.”
Topics provoking harassment		
Masking	24	“After advocating for masking in our local schools, photographs were taken without my consent at an outdoor pool setting of myself in my bathing suit and my daughter in just a diaper. These were posted all over social media.”
Vaccinations	41	“I have had some of the most angry, vile messages sent to me. I have had someone suggest I should be followed and potentially harmed for assisting with state COVID contact tracing and vaccination efforts.”
		“When I posted a picture of myself with my badge in my white coat after my COVID-19 vaccination I received hundreds of harassing anti-vaxx messages including death threats.”
Public health	50	“It became clear that public-facing physicians had a small but real responsibility to lend their credentials to support public health guidance, as well as periodically to use specific subject matter expertise when appropriate.”
		“As a pediatric infectious disease physician, I felt it important to serve as a trusted account for reliable information about the pandemic and especially its impact on children.”
Abortion advocacy	11	“I am an abortion provider and regularly receive threats of violence on my social media. People on social media have threatened my safety, my life and my family’s safety.”
		“I advocate for unfettered access to abortion care and for this, I have had death threats. I have consistent harassment on social media, and this has resulted in severe consequences to my career.”
Firearm safety	7	“Have been harassed primarily around injury prevention messaging, including firearm storage and helmet usage in bicycling.”
		“Have been encouraged to commit suicide during a debate on gun violence and restrictions.”
Transgender advocacy	6	“The harassment happens mostly when posting about racial inequity, transgender issues, and COVID-19.”
Race	10	“Numerous anti-Asian racist attacks plying racist tropes.”
		“Prior to the pandemic social media was just me goofing off. As misinformation grew, I found myself consistently tweeting out information or retweeting tweets to correct misinformation. Of course, that led to increased harassment, especially as a Black woman scientist.”
Religion	4	“Harassment tended to be in the form of negative patient reviews, emails with anonymized email addresses, and hate directed at me for identifying as Muslim.”
		“Death threats. Insults. Antisemitic threats.”

Abbreviation: DMs, direct messages.

^a^ Other advocacy topics that resulted in harassment included substance use disorders (3 responses), race and racism (3 responses), LGBTQ issues (3 responses), obesity (2 responses), bike helmets (1 response), medical assistance in dying (1 response), environmental justice (1 response), Ukraine war (1 response), breastfeeding (1 response), scope of practice (1 response), and child psychiatry (1 response).

^b^ Doxxing refers to the act of publishing private or identifying information about an individual on the internet, typically with malicious intent.

## Discussion

Physicians and biomedical scientists experience high levels of harassment online, a problem that appears to have been worse during the COVID-19 pandemic.^1^ Social media plays a role in disseminating medical and scientific knowledge to the public; however, high levels of reported harassment may lead more physicians and scientists to limit the way they use social media, thus leaving propagation of misinformation unchecked by those most qualified to combat it.

Study limitations include potential nonresponse bias, self-selection bias, and recall bias, as participants were asked to recall any instances of harassment, whether remote or recent. At a time when physicians and biomedical scientists need support and their advocacy is vital to the national interest more than ever before, they are being badgered, doxxed, and sexually harassed. Institutions and companies should support those who are attacked and provide mechanisms to reduce harassment and provide accountability.

## Notes

Supplement 1. **eMethods.** Default Question Block **eFigure.** Recruitment Figure Used on Twitter

Supplement 2.
Data Sharing Statement
