# Supplementary material for: Physician and Biomedical Scientist Harassment on Social Media During the COVID-19 Pandemic
Source: JAMA Netw Open. 2023 Jun 14;6(6):e2318315. doi: 10.1001/jamanetworkopen.2023.18315 (PMC10267768; doi:10.1001/jamanetworkopen.2023.18315)
Supplement: Supplement 1. — eMethods. Default Question Block eFigure. Recruitment Figure Used on Twitter [file jamanetwopen-e2318315-s001.pdf]

Supplemental Online Content

Royan R, Pendergrast TR, Woitowich NC, et al. Physician and biomedical scientist harassment on social media during the COVID-19 pandemic. *JAMA Netw Open.* 2023;6(6):e2318315.
doi:10.1001/jamanetworkopen.2023.18315

eMethods. Default Question Block
eFigure. Recruitment Figure Used on Twitter

This supplemental material has been provided by the authors to give readers additional information about their work.

© 2023 Royan R et al. *JAMA Network Open.*

Northwestern | QUALTRICS

Default Question Block

Title of Research Study: Social Media Harassment of Physician Women and Minority Groups - A Follow Up Study
IRB Study Number: STU00216831
Principal Investigator: Mary Royan, MD
Supported By: This research is supported by the Department of Emergency Medicine at Feinberg University School of Medicine.

Key Information about this research study: The purpose of this study is to learn more about physicians' and medical trainees' use of social media. We are interested in learning about the prevalence, and experiences related to, harassment on social media. We want to understand if certain groups are targeted with online harassment more than others. You will be asked to fill out an anonymous survey one time. We expect that you will be in this research study for ten to fifteen minutes.

© 2023 Royan R et al. *JAMA Network Open.*

Why am I being asked to take part in this research study?

The inclusion criteria for this study is that you are able to speak English and reside in the United States AND you are a physician, scientist, or in training to become a physician or scientist. You are seeing this consent form because you clicked on a link that brought you to the study page. You can close out of the survey at any time. Your participation will help us answer the objective of our study which is to better understand harassment of physicians on social media and whether this harassment affects certain groups more than others. We are also interested in learning if this harassment has changed during the coronavirus pandemic or is affected by being verified.

How many people will be in this study?

We expect about 500 people total to participate in this study.

What should I know about a research study?

- Whether or not you take part is up to you.
- You can choose not to take part.
- You can agree to take part and later change your mind.
- Your decision will not be held against you.

If you say that “Yes, you want to be in this research,” here is what you will do

Click “Yes” below. You will be prompted to fill out a survey one time.

Will being in this study help me in any way?

There are no direct benefits to you.

What happens if I do not want to be in this research or if I say “Yes”, but I change my mind later?

Participation in research is voluntary. You can decide to participate or not to participate. You may skip any questions that make you feel uncomfortable. You can decide not to participate in this research or you can start and then decide to leave the research at any time and it will not be held against you. To do so, simply exit the survey. Any data already collected will not be saved.

What happens to the information collected for the research?

This survey is being hosted by Qualtrics and involves a secure connection. Terms of service, addressing confidentiality, may be viewed at: https://www.qualtrics.com/terms-of-service/

© 2023 Royan R et al. *JAMA Network Open.*

Data Sharing

This survey is anonymous, but there is a free-text section. If you provide any identifying information in your responses, we will not include any identifying information in any publications or presentations about this study, and any such identifying information will not be included in the data if they are shared with other researchers. De-identified data from this study may be shared with the research community at large to advance science and health. We will remove or code any personal information that could identify you before files are shared with other researchers to ensure that, by current scientific standards and known methods, no one will be able to identify you from the information we share. Despite these measures, we cannot guarantee anonymity of your personal data.

Who can I talk to? If you have questions, concerns, or complaints talk to the co-investigator Tricia Pendergrast (tricia.pendergrast@northwestern.edu).

This research has been reviewed and approved by an Institutional Review Board ("IRB"). You may talk to them at (312) 503-9338 or email irb@northwestern.edu if:

- Your questions, concerns, or complaints are not being answered by the research team.
- You cannot reach the research team.
- You want to talk to someone besides the research team.

© 2023 Royan R et al. *JAMA Network Open.*

• You have questions about your rights as a research participant.
• You want to get information or provide input about this research.

Consent

If you want a copy of this consent for your records, you can print it from the screen.

If you wish to participate, please click "Yes" next to the I Agree text, and you will be taken to the survey. If you do not wish to participate in this study, please select "No" or select "X" in the corner of your browser.

I Agree:

- ○ Yes
- ○ No

Inclusion Criteria

Please select your primary role:

- ○ I am a physician, medical student or trainee.
- ○ I am a biomedical scientist, graduate student, postdoctoral fellow or trainee
- ○ None of the above

This survey is for physicians **and** biomedical scientists. However, some individuals may have dual-degrees.

Are you a physician-scientist [MD, PhD] or a physician-scientist in training)?

○ Yes
○ No

Do you currently reside in the United States?

○ Yes
○ No

Demographic Information

In which state do you currently reside?

© 2023 Royan R et al. *JAMA Network Open.*

Please select your age range:

- ○ 18 – 24 years old
- ○ 25 – 34 years old
- ○ 35 – 44 years old
- ○ 45 – 54 years old
- ○ 55 – 64 years old
- ○ 65 – 74 years old
- ○ 75 years or older
- ○ Prefer not to say

Which group most accurately describes your race?

- ○ American Indian or Alaskan Native
- ○ Asian
- ○ Black or African American
- ○ Native Hawaiian or other Pacific Islander
- ○ White
- ○ Multiracial
- ○ Some other race or origin
- ○ [ ] Prefer to self-describe
- ○ Prefer not to say

© 2023 Royan R et al. *JAMA Network Open.*

Are you of Hispanic, Latino/a/x, or of Spanish origin?

- Yes
- No
- Prefer not to say

What is your current gender identity?

- Man
- Woman
- Trans male / Trans man
- Trans female / Trans woman
- Gender non-binary or non-conforming
- Prefer to self-describe
- Prefer not to say

What is your current relationship status?

- Single
- Married
- Partnered
- Divorced
- Widowed
- [ ] Something else [describe below]
- Prefer not to say

© 2023 Royan R et al. *JAMA Network Open.*

Do you identify as disabled?

- ○ Yes
- ○ No
- ○ Prefer not to say

Do you identify as being a minority on the basis of gender or sexuality?

- ○ Yes
- ○ No
- ○ Prefer not to say

Please select degrees you've obtained (check all that apply):

- ☐ BS or BA
- ☐ MS or MA
- ☐ MPH
- ☐ MD, DO, or MBBS
- ☐ PhD
- ☐ Other international degree [describe below]: ______
- ☐ Other professional degree [describe below]: ______

© 2023 Royan R et al. *JAMA Network Open.*

Education & Work Environment

Are you currently a medical student, resident, fellow, or other trainee?

- ○ No
- ○ Yes, I am a medical student
- ○ Yes, I am a resident
- ○ Yes, I am a fellow
- ○ Yes, I am a trainee (other)

Are you currently a graduate student or postdoctoral fellow?

- ○ No
- ○ Yes, I am a graduate student
- ○ Yes, I am a postdoctoral fellow

Please select the speciality you primarily practice in from the drop-down list below:

© 2023 Royan R et al. *JAMA Network Open.*

Please select your primary field of research or study:

[ ]

Please select the number of years in practice after completing residency:

- ○ 0 – 5 years
- ○ 6 – 10 years
- ○ 11 - 15 years
- ○ 16 – 20 years
- ○ More than 20 years

Please select the number of years since completing your PhD:

- ○ 0 –5 years
- ○ 6 – 10 years
- ○ 11 - 15 years
- ○ 16 – 20 years
- ○ More than 20 years

© 2023 Royan R et al. *JAMA Network Open.*

Please select your primary work environment:

- ○ Clinical care
- ○ Research
- ○ Education
- ○ Administration (ie. division chief, department chair, medical director, dean, provost)
- ○ Industry
- ○ Government or non-profit
- ○ Other

Do you hold an academic faculty position?

- ○ Yes
- ○ No

What is your current faculty rank?

- ○ Assistant Professor
- ○ Associate Professor
- ○ Professor
- ○ Instructor

© 2023 Royan R et al. *JAMA Network Open.*

Social Media Use

The next series of questions relates to your use of social media platforms or applications.

Which of the following social media platforms do you currently use?

	Yes	No
Facebook	○	
○ Twitter	○	
○ Instagram	○	
○ TikTok	○	
○ YouTube	○	
○ Other [ ]	○	○

© 2023 Royan R et al. *JAMA Network Open.*

Do you use your legal (given) name on your social media account(s)?

- ○ Yes, I use my full legal name (eg. John Smith)
- ○ Yes, I use part of my legal name (eg. John S.)
- ○ No, I use an alias, pseudonym, or screen name.

Do you list your school, employer or institutional affiliation(s) on your social media account(s)?

- ○ Yes
- ○ No

Social Media Harassment

Content Warning: Please note that the next series of questions contain content regarding sexual and other forms of harassment.

Have you ever been harassed on social media?

- Yes
- No
- Unsure
- Prefer not to say

On social media, have you ever been harassed on the basis of your:

	Yes	No
Gender	○	
○ Race or ethnicity	○	
○ Sexual orientation	○	
○ Disability status	○	
○ Advocacy work	○	
○		
For another reason not listed here [describe below]	○	○

© 2023 Royan R et al. *JAMA Network Open.*

On social media, have you ever been harassed in relation or regards to the Covid-19 pandemic?

- Yes
- No
- Unsure
- Prefer not to say

Have you ever been sexually harassed on social media (ex. sexual commentary, advancements, lewd images etc.)?

- Yes
- No
- Unsure
- Prefer not to say

© 2023 Royan R et al. *JAMA Network Open.*

Has your private information (phone number, address, employment information) ever been posted online with malicious intent (doxing)?

- Yes
- No
- Unsure
- Prefer not to say

Covid-19 Pandemic

The next series of questions relates to your use of social media during the Covid-19 pandemic.

Did you use social media prior to the start of the Covid-19 pandemic?

- Yes
- No

© 2023 Royan R et al. *JAMA Network Open.*

Has the Covid-19 pandemic changed the way in which you use social media?

- Yes
- No
- Unsure

Do you use social media to post public health messages or content (encouraging mask wearing, vaccinations, social distancing, etc.)

- Yes
- No
- Unsure

Verification

The next series of questions is about social media verification.

© 2023 Royan R et al. *JAMA Network Open.*

Have you ever applied to be verified on social media?

- ○ Yes
- ○ No
- ○ Unsure

Are you currently verified on social media?

- ○ Yes
- ○ No

Which social media platforms are you currently verified on? Please select all that apply:

- ☐ TikTok
- ☐ Facebook
- ☐ Twitter
- ☐ Instagram
- ☐ [ ] Other

© 2023 Royan R et al. *JAMA Network Open.*

Did it take multiple attempts for you to become verified on social media?

- ◯ Yes
- ◯ No

Free Response

You indicated that you have been harassed on social media. Would you like to share more about your experiences?

- ◯ No
- ◯ Yes

You indicated that the Covid-19 pandemic has changed how you use social media. Would you be willing to share more about you experiences?

© 2023 Royan R et al. *JAMA Network Open.*

○ No

○ Yes

You indicated that you have been verified on one or more social media platforms. Would you be willing to describe if or how that has impacted you?

○ No

○ Yes

© 2023 Royan R et al. *JAMA Network Open.*

[Optional]

If you have further comments you would like to share about your online experiences or about your participation in this study, please provide them below:

Powered by Qualtrics

© 2023 Royan R et al. *JAMA Network Open.*

eFigure. Recruitment Figure Used on Twitter

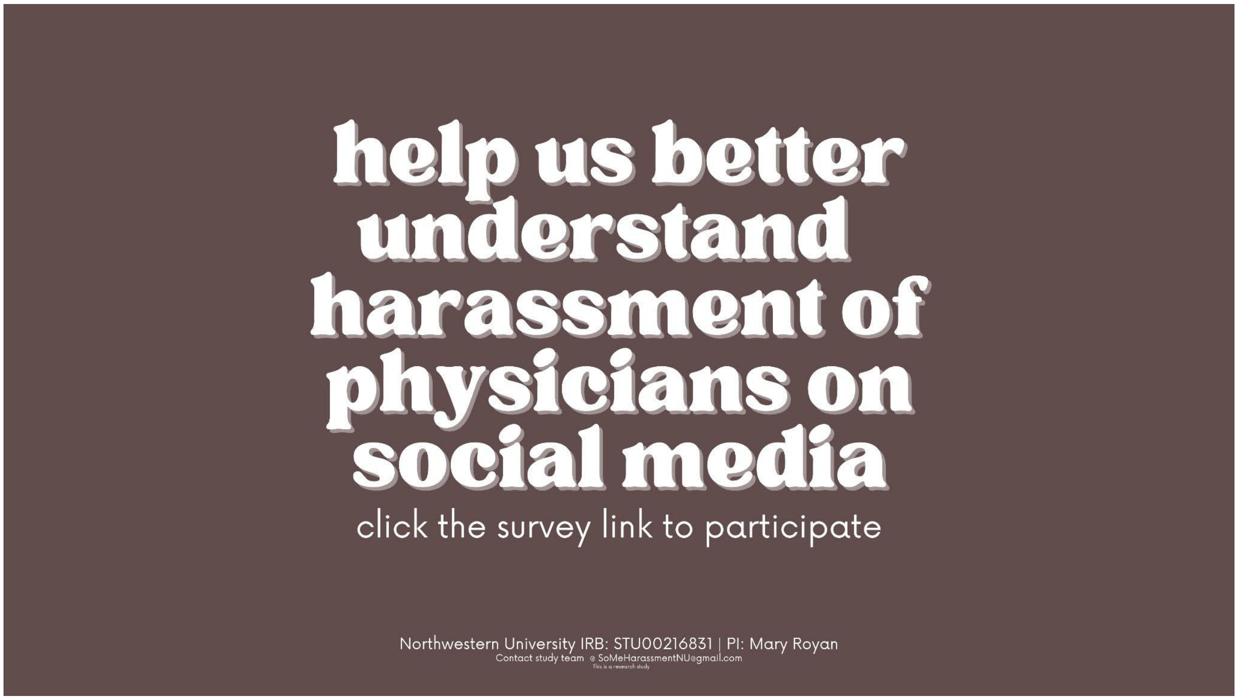

© 2023 Royan R et al. *JAMA Network Open.*
