# Supplementary material for: Physician and Biomedical Scientist Harassment on Social Media During the COVID-19 Pandemic
Source: JAMA Netw Open. 2023 Jun 14;6(6):e2318315. doi: 10.1001/jamanetworkopen.2023.18315 (PMC10267768; doi:10.1001/jamanetworkopen.2023.18315)
Supplement: Supplement 2. — Data Sharing Statement [file jamanetwopen-e2318315-s002.pdf]

Data Sharing Statement

Royan. Physician and Biomedical Scientist Harassment on Social Media During the COVID-19 Pandemic. *JAMA Netw Open*. Published June 14, 2023.
doi:10.1001/jamanetworkopen.2023.18315

Data
Data available: Yes
Data types: Deidentified participant data
How to access data: Upon reasonable request of the authors.
When available: With publication

Supporting Documents
Document types: None

Additional Information
Who can access the data: Researchers whose proposed use of the data has been approved.
Types of analyses: Any
Mechanisms of data availability: with investigator support after approval of proposal.
